# Incidence and Survival Outcomes in Patients with Lung Neuroendocrine Neoplasms in the United States

**DOI:** 10.3390/cancers13081753

**Published:** 2021-04-07

**Authors:** Shrunjal Shah, Rohit Gosain, Adrienne Groman, Rahul Gosain, Arvind Dasari, Thorvardur R. Halfdanarson, Sarbajit Mukherjee

**Affiliations:** 1 Department of Medicine/Hematology & Medical Oncology, Roswell Park Comprehensive Cancer Center, Buffalo, NY 14263, USA; shrunjal.shah@roswellpark.org; 2 Department of Medical Oncology and Hematology, UPMC Hillman Cancer Center, UPMC Chautauqua, 207 Foote Avenue, Jamestown, NY 14701, USA; gosainr@upmc.edu; 3 Department of Biostatistics and Bioinformatics, Roswell Park Comprehensive Cancer Center, Buffalo, NY 14263, USA; adrienne.groman@roswellpark.org; 4 Department of Medical Oncology and Hematology, Guthrie Corning Cancer Center, Corning, NY 14830, USA; rahul.gosain@guthrie.org; 5 Department of GI Medical Oncology, Division of Cancer Medicine, The University of Texas MD Anderson Cancer Center, Houston, TX 77030, USA; adasari@mdanderson.org; 6 Department of Oncology, Division of Medical Oncology, Mayo Clinic College of Medicine, Rochester, MN 55902, USA; halfdanarson.thorvardur@mayo.edu

**Keywords:** neuroendocrine neoplasm, neuroendocrine tumors, bronchial neuroendocrine tumors, incidence, survival, prognosis, epidemiology, atypical carcinoid, typical carcinoid, small cell lung cancer, large cell lung carcinoma, pulmonary neuroendocrine tumors, SEER1

## Abstract

**Simple Summary:**

Neuroendocrine tumors are a rare group of neoplasms characterized by strikingly heterogeneous pathological features and clinical behavior. The incidence and prevalence of these tumors are rising. Studies have reported the incidence and prevalence of neuroendocrine tumors; however, specific data targeting lung neuroendocrine tumors are lacking. We conducted a retrospective analysis using a national database to report the incidence and survival trends of lung neuroendocrine neoplasms. We found that the overall survival and disease-specific survival trends varied significantly not only by stage and histological type but also by age, race, marital status, and insurance type. A small difference was also noted between the rural and urban populations. The rarity of these tumors poses several diagnostic and therapeutic challenges. Our findings generate the following hypothesis that increased awareness of these tumors, as well as better diagnostic modalities, may contribute to a higher incidence. Furthermore, newer therapeutic advances may have caused an improvement in the survival trends in recent years. Such population-based analysis is important to allot resources and guide future research in the field.

**Abstract:**

Background: The incidence and prevalence of neuroendocrine neoplasms (NENs) are rapidly rising. Epidemiologic trends have been reported for common NENs, but specific data for lung NENs are lacking. Methods: We conducted a retrospective analysis utilizing the Surveillance, Epidemiology, and End Results (SEER) database. Associated population data were utilized to report the annual age-adjusted incidence and overall survival (OS) trends. Trends for large-cell neuroendocrine carcinoma (LCNEC) and atypical carcinoid (AC) were reported from 2000–2015, while those for typical carcinoid (TC) and small cell lung cancer (SCLC) were reported from 1988–2015. Results: We examined a total of 124,969 lung NENs [103,890—SCLC; 3303—LCNEC; 8146—TC; 656—AC; 8974—Other]. The age-adjusted incidence rate revealed a decline in SCLC from 8.6 in 1988 to 5.3 in 2015 per 100,000; while other NENs showed an increase: TC increased from 0.57 in 1988 to 0.77 in 2015, AC increased from 0.17 in 2001 to 0.22 in 2015, and LCNEC increased from 0.16 in 2000 to 0.41 in 2015. The 5-year OS rate among SCLC, LCNEC, AC, and TC patients was 5%, 17%, 64%, and 84%, respectively. On multivariable analyses, OS and disease-specific survival (DSS) varied significantly by stage, sex, histological type, insurance type, marital status, and race, with a better survival noted in earlier stages, females, married, insured, Hispanic and other races, and urban population. Similarly, TC and AC had better survival compared to SCLC and LCNEC. Conclusion: The incidence of lung NENs is rising, possibly in part because of advanced radiological techniques. However, the incidence of SCLCs is waning, likely because of declining smoking habits. Such population-based studies are essential for resource allocation and to prioritize future research directions.

## 1. Introduction

Neuroendocrine tumors (NENs) are a rare set of tumors which originate from neuroendocrine cells in the body. Overall, neuroendocrine tumors comprise of 0.5–2% of all malignancies occurring in adulthood [1]. Pulmonary NENs are even a rare set amongst those that account for approximately 20–25% of all invasive lung malignancies, whereas 75% comprises of non-small cell lung cancer (NSCLC) [2,3,4,5]. Pulmonary NENs are classified into four main categories, as described by the WHO [6]; namely typical carcinoid (TC), atypical carcinoid (AC), large cell neuroendocrine carcinoma (LCNEC), and small cell lung carcinoma (SCLC). It is extremely important to establish a clear diagnosis early on as the diagnosis further has important treatment and prognostic implications. For several decades, these tumors were considered benign and were excluded from the cancer registries. A seminal study in 2008 by Yao at al. [7] describing the rising incidence and prevalence of neuroendocrine tumors placed NEN in the spotlight. 

The incidence and prevalence of NENs continue to rise in a linear pattern [8,9]. This increase in incidence could be from advanced radiological techniques along with better understanding of this disease leading to better diagnosis [8,9]. Given the rarity of these tumors, it is important to evaluate the incidence and survival outcomes retrospectively using large population databases. The Surveillance, Epidemiology, and End Results (SEER) program is an example of such database to gather population-based information [10]. Dasari et al. evaluated the incidence, prevalence, and survival outcomes of patients with neuroendocrine tumors in the United States, demonstrating a linear pattern of increasing NEN patients; however, they did not specifically report lung NEN outcomes [8]. 

To help understand the lung NEN incidence and survival outcomes, we analyzed the SEER database in this retrospective study. 

Little is known about the causative etiologies and risk factors. The heterogeneity of NENs makes the diagnosis challenging, and due to the rarity, there is limited consensus in treatment guidelines amongst the various societies. To complicate matters more, variations in socioeconomic status, marital status, and insurance coverage are also known to cause discrepancies in outcomes. Our study not only highlights the incidence and survival trends, but also sheds light on the differences in outcomes based on such variables. The advent of novel treatment modalities presents new challenges, emphasizing the need for prospectively designed clinical trials. 

In light of these, such epidemiological studies are sure to remain a cornerstone in creating awareness and allocating resources. 

## 2. Methods

### 2.1. Data Source

To evaluate lung NEN incidence and survival outcomes retrospectively, we utilized the SEER program and evaluated data from years 1988–2015. The SEER program is a population-based database established by the National Cancer Institute in 1973, which is updated annually. The SEER database collects cancer-specific incidence data from population-based registries, including about 35% of the United States (US) population [10]. The histological type of the tumor, stage at diagnosis, and survival data are some of the key components that are also captured by the database. Additionally, the patient demographics in SEER database closely resemble that of the US population. Since SEER’s program establishment in 1973, the program has undergone two major expansions, SEER 13 in 1992 and SEER 18 in 2000, this was to cover 20 additional geographic areas. 

### 2.2. Study Population

NEN patients of all ages, with any analytical stage I, II, III, IV were included. Pulmonary NENs are classified into 4 main categories: SCLC, LCNEC, TC, and AC. Initially in 1970, only SCLC, TC, and AC were considered to be pulmonary NENs [11,12]. However, in the fourth edition of the World Health Organization (WHO) LCNEC definition was redefined and was further included in pulmonary NEN following 1999 [13]. 

To obtain appropriate data from the SEER database, we utilized diagnostic codes from the International Classification of Diseases for Oncology, 3^rd^ edition (ICD-O-3). Patients with the following ICD histologic codes were included: SCLC, LCNEC, TC, AC, and unknown lung NENs (ICD-O codes 8002, 8040–8045; 8013; 8240; 8249; 8246, respectively). Additionally, we compared the survival of different lung NENS with adenocarcinoma, the most common form of lung cancer. 

### 2.3. Statistical Analysis

Patient demographic and clinical characteristics were summarized by histology using means, medians, and standard deviations for the continuous variables and frequencies and cumulative frequencies for categorical data. Comparisons were made using the Kruskal–Wallis test for continuous variables or Pearson chi-square tests for categorical variables. Overall Survival (OS) and disease-specific survival (DSS) were summarized by using standard Kaplan–Meier methods, where estimates of the median OS and DSS were obtained with 95% confidence intervals (CIs). OS was defined by the time from diagnosis to death due to any cause. DSS was defined by the time from the diagnosis to death due to cancer itself. Comparisons were made using the log-rank test. Univariate and multivariable Cox proportional hazard models (adjusting for age, gender, race, insurance status, histological type, stage at the time of diagnosis, area of residence, and marital status) were used to estimate the effects of different covariates on both OS and DSS in study participants. Result estimates were expressed as hazard ratios (HR) with 95% confidence intervals (CI). Subgroup analyses were conducted on each histological type to examine the effect of covariates on survival within histology. Incidence rates were calculated for each histology and SEER historical stage over time using the SEER population database. All analyses were conducted in SAS v9.4 (Cary, NC) at a significance level of 0.05. 

## 3. Results

We examined a total of 124,969 lung NENs. Of this patient population, the majority was SCLC (103,890—83%) patients, followed by TC (8146—6%), LCNEC (3303—3%) and AC (656—0.5%). Additionally, a significant portion was NENs not-classified in any category and reported as “others” (8974—7%). Median age for aggressive tumors, SCLC and LCNEC, was 67 (15–99) years while the less aggressive tumor types, TC and AC, had slightly lower median age at 60 (10–96) years. Demographics of lung NEN patients is summarized in Table 1 below. 

Majority of the NENs have seen an uptrend in number of cases in the past decade [6]. When evaluating lung NENs, we did not observe a uniform scenario, but rather a mixed trend. SCLC age-adjusted annual incidence rate per 100,000 people in 1988 was 8.6, and with gradual decline over the years. In 2015 it was recorded to be 5.3. 

**Figure 1:**
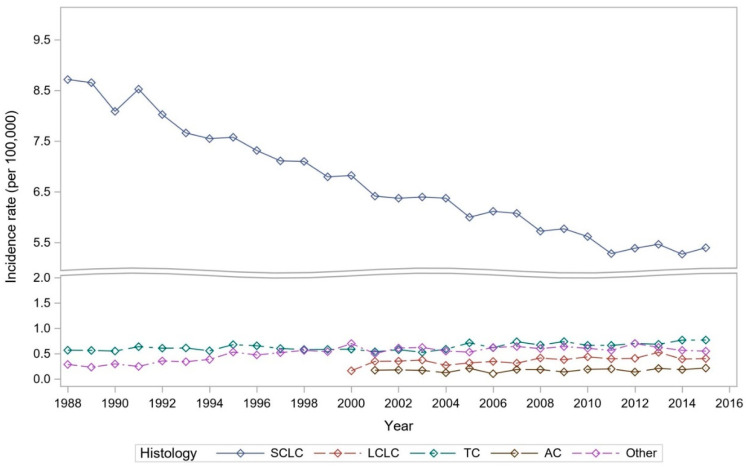
Incidence trends over the years among lung cancer patients with different histology (small cell lung cancer (SCLC) vs. typical carcinoid (TC), atypical carcinoid (AC), large-cell neuroendocrine carcinoma (LCNEC) and others).

LCNEC was initially reported in 2000. Incidence recorded in 2000 was 0.16, whereas in 2015 its incidence had more than doubled at 0.41. On the other hand, less aggressive tumor types TC have also shown uptrend in the incidence with 0.57 in 1988 and 0.77. The rarest type, AC, when initially reported in 2001 was 0.17, and in 2015 incidence was slightly increased at 0.22. Figure 1 further depicts these trends in the lung NENs over the years. 

**Figure 2:**
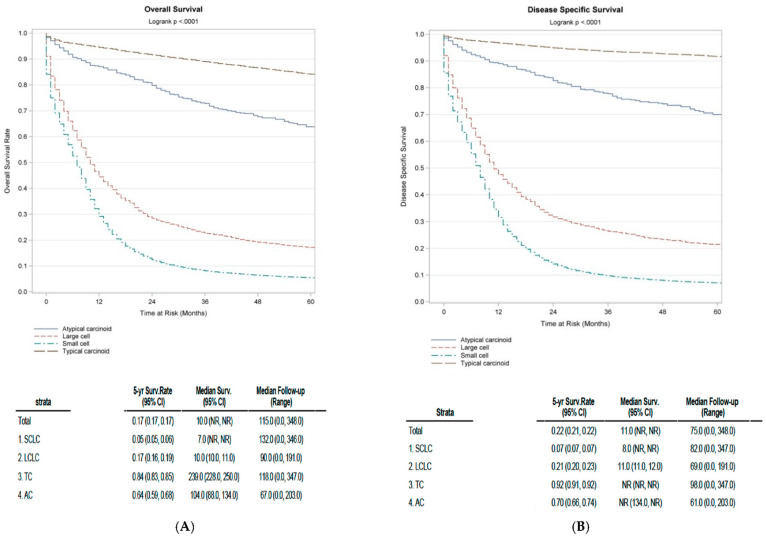
(**A**) Unadjusted Kaplan-Meier curves depicting median overall survival (OS) of lung cancer patients based on different histological type. (**B**) Unadjusted Kaplan-Meier curves depicting median disease-specific survival (DSS) of lung cancer patients based on different histological type.

The median overall survival (OS) for all lung cancer patients was 10 (95% CI: NR, NR) months. Among these, patients with localized lung cancer had the best median OS (54 months), followed by patients with regional spread lung cancer (17 months), with worst outcomes observed in patients with distant metastasis (5 months). When focusing specifically on lung NEN survival, patients with SCLC had the least median OS: 7 (NR, NR) months. Interestingly, there was a continuous improvement in overall survival trend for SCLC and TC until 2002, however this effect seemed to plateau thereafter, as seen in Supplementary Table S1A,C. LCNEC followed SCLC with a median OS of 10 (10.0, 11.0) months. Patients with AC had a median OS of 104 (88.0, 134.0) months, whereas those diagnosed with TC had the most favorable median OS of 239 (228.0, 250.0) months. The 5-year OS rate among SCLC and LCNEC patients was 5% and 17%, respectively, consistent with their aggressive nature. On the other hand, TC and AC, representing more indolent NENs, had a better 5-year OS rate of 84% and 64%, respectively (Figure 2). 

**Figure 3:**
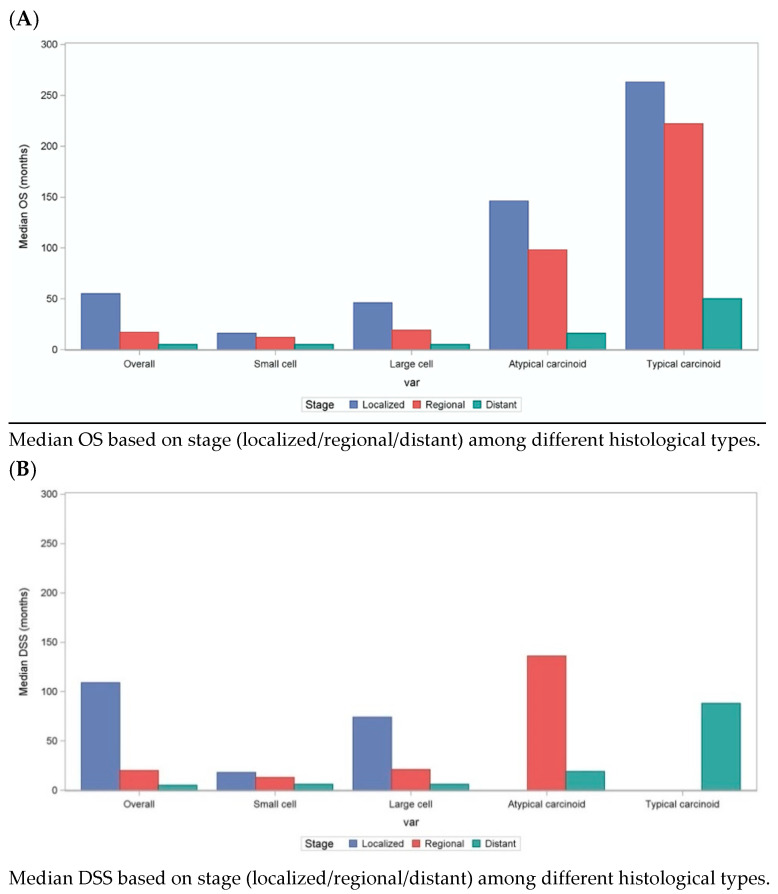
(**A**) Median OS among lung cancer patients based on different stages among different histological type. (**B**) Median DSS among lung cancer patients based on different stages among different histological type. On multivariable analysis, the median overall survival (OS) and disease-specific survival (DSS) rate varied significantly by stage, age at diagnosis, histological type, insurance type, marital status, and race.

Among all lung NENs, localized patients had better OS (SCLC—16 months, LCNEC—46 months, AC—146 months, TC—263 months; *p* < 0.01), when compared to regional (SCLC—12 months, LCNEC—19 months, AC—98 months, TC—222 months; *p* < 0.01) and metastatic disease (SCLC—5 months, LCNEC—5 months, AC—16 months, TC—50 months; *p* < 0.01). Similar trends were observed when comparing DSS among different lung NENs. Irrespective of the stage, patients with SCLC and LCNEC had poor DSS: median DSS of 8 (NR, NR) months vs. 11(11.0, 12.0) months, respectively, when compared to TC and AC patients (not reached). These results are further summarized in Figure 3 below.

OS and DSS HR for distant vs. localized disease was 3.24 (CI 3.21–3.28) and 4.12 (CI 4.02–4.17), respectively. OS and DSS HR for female vs. male was 0.81 (CI 0.80–0.81) and 0.83 (CI 0.82–0.83), respectively. The OS and DSS HR for single vs. married individuals was 1.17 (CI 1.17–1.18) and 1.15 (CI 1.15–1.16), respectively. OS and DSS HR for uninsured vs. insured individuals was 1.22(CI 1.19–1.25) and 1.19 (CI 1.16–1.23). It appears that race did not impact survival. The Hispanic population had a marginally better survival compared to Non Hispanic White(NHW), with OS and DSS 0.97 (CI 0.96–0.98) and 0.96 (CI 0.95–0.97), respectively. There was no OS and DSS difference noted between NWH and Non Hispanic Black(NWB), however other races seemed to have better OS and DSS in comparison to NHW (0.80 (CI 0.79–0.81) and 0.79 (CI 0.78–0.81), respectively.

To summarize, typically Stage I, localized disease, female gender, insured status, married, urban residence patients had better OS and DSS when compared to stage II/III/IV, regional/metastatic disease, male gender, uninsured status, single and rural residence patients, respectively. When comparing OS and DSS of different histological types of lung NENs to lung adenocarcinoma, patients with TC and AC had better OS and DSS. However, patients with LCNEC and SCLC had inferior outcomes when compared to adenocarcinoma. These results are further summarized in Table 2 below. Further, we evaluated OS among lung NEN patients specifically, based on 5-year intervals, results outlined in Table S1A–D. This analysis showed better OS in the most recent years among TC and SCLC patients in comparison to earlier set of years.

## 4. Discussion

NENs originate in neuroendocrine cells and can affect almost any part of the body. Although NENs only comprise about 0.5–2% of all malignancies in adults, there has been almost a 7-fold increase in the last decade. Dasari et al. reported that the incidence of NENs had risen from 1 in 100,000 population in the 1970s to 6.98 in 100,000 in 2012. They also reported a marked increase in the 20-year limited duration prevalence, estimated to be 171,321 in 2017 as compared to 103,312 in 2004 [8]

However, to date, there have been limited studies that specifically aimed at evaluating the trends in incidence, prevalence, and OS of lung NENs. One such population-based study performed in Demark reported trends in demographics, incidence, and survival of patients with pulmonary NENs from 1978–1997. They saw a similar increasing incidence of pulmonary NENs except for SCLCs, although that study had a much smaller sample size. [14]. Another study by Petursdottir et al. reported a mean increase of 29% of pulmonary neuroendocrine tumors in the Icelandic population per decade of the study period (1955–2015). [15] 

We found that with the exception of SCLC, the age adjusted annual incidence of lung NENs has steadily been increasing. SCLC is strongly associated with cigarette smoking. The decrease in the incidence of SCLC can be attributed to several factors, including the decreased percentage of smokers, change in cigarette composition, use of filters, as well as reduced occupational hazards [16]. However, this downtrend in incidence has seemed to plateau since 2002. Factors contributing to this effect could be stage migration and the fact that previously diagnosed borderline mixed histological subtypes are now classified as non-small cell lung cancer. Stage migration, also known as the “Will Roger’s phenomenon”, could have also occurred owing to the improved radiological detection of NENs. Furthermore, updated staging and grading classifications for NENs have been validated, increasing the recognition of NENs. The association of smoking with other lung NENs is much less known. Therefore, owing to a multitude of factors including advanced diagnostic techniques and more accurate staging, the rates of diagnosis have significantly improved, potentially explaining some of the observed increase in incidence over time. 

Our SEER-based results showed that the median age at diagnosis was lower for TC and AC as compared to the more aggressive tumors. Another interesting finding was the increased incidence of TC and AC in females (67% and 64%, respectively, vs. 33% and 36% in males). The etiology remains unclear; however, Annette Fisseler-Eckhoff described a particular type of neuroendocrine cell hyperplasia known as “tumorlets” (preinvasive lesions) that were predominantly found in females [17].

The average 5-year survival rate in the reviewed literature for TC and AC is 93% (range 88–97%) and 69% (range 40–86%), respectively. In our study, the overall survival rates for TC and AC were 84% and 64%, respectively. We believe that this reported difference is related to the heterogeneous presentation, as well as treatment of lung NENs. The mainstay of treatment for localized disease is surgical resection. The choice of surgery (lobectomy vs. segmentectomy), systematic nodal dissection differs from institution to institution, with possibly better outcomes at high volume centers [18]. Our data are more likely to represent the real-world scenario due to a large number of cases from different institutions across the country.

Variations in socioeconomic status, insurance coverage, and access to health care have been known to cause disparities in clinical outcomes. Our multivariable analysis revealed that insured patients tend to have better OS when compared to uninsured patients. A marginal difference was noted between the rural and urban populations as well. 

Lack of awareness and limited access to tertiary care centers could be potential contributing factors. In a recent study by Gosain et al. [19], there was a higher incidence of NEN in rural areas along with an increased proportion of advanced stages at the initial time of presentation. This highlights the need for redirection of healthcare resources and changes in healthcare policies to address these disparities and improve outcomes. 

Another notable difference in OS and DSS was between single and married individuals. Marital status has been proven to be an important prognostic factor in several malignancies, including breast, prostate, and colorectal carcinoma. Studies have shown an association between married status and earlier stages at the time of diagnosis, pursuant of definitive rather than expectant treatment, improved compliance, and overall psychological health [20]. Similar findings in survival from our multivariate analysis only accentuate this disparity. 

Finally, although the incidence has been steadily increasing, there has been a steady improvement in the overall survival since 2002. This was notable only for TC and SCLC, likely in lieu of earlier detection and novel treatment strategies. 

## 5. Limitations

While the SEER database is an extremely valuable tool for research, certain limitations must be considered when interpreting results from a SEER observational study. The reliability in coding for rare malignancies such as NENs can be variable, especially if not considered malignant. Furthermore, there is no quality assurance regarding pathology interpretation. This can falsely underreport the true incidence and prevalence. Factors like migration (patients moving from one SEER registry region to another) can cause loss of information from the database, difficulty with long-term follow up and less reliable measurements of outcomes. The SEER database is also unable to capture important factors determining treatment decisions such as lifestyle factors, symptom burden at diagnosis, time to diagnosis etc., as well as quality of treatment such as the details of treatment received. Other limitations that also need to be taken into consideration revolve around unrecorded prognostic variables such as co-morbidities, tumor size, or margins at the time of surgery, all of which could potentially play as confounding factors. 

The large size and longitudinal follow up of our study provide a comprehensive review of the demographics and outcomes of lung NENs and requites some of the limitations intrinsic to any population-based retrospective study. 

## 6. Future Directions

Surgery remains the mainstay of treatment for localized lung neuroendocrine tumors. Given the indolent behavior of TC, adjuvant therapy seems to confer no survival benefit. On the contrary, AC should be treated with a multimodality approach, given their propensity to metastasize, conferring to higher relapse rates and poor overall survival [21]. Treatment recommendations for adjuvant systemic therapy and radiation differ amongst different guidelines (NCCN, ENETs, NANETs) [22]. Treatment decisions are usually made in the multi-disciplinary setting on an individual basis. 

A small proportion of patients with lung NENs will present in the advanced/metastatic setting. Outside of the conventional treatment options, newer exciting treatment modalities have come into play. Peptide Receptor Nucleotide therapy (PRRT) and molecular targeted therapy have broadened the landscape of treatment options. With mTOR inhibitor Everolimus receiving a FDA approval in 2016, the doors to targeted therapies have opened for lung NENs. Genomic profiling has shown that LCNEC carries a high tumor mutational burden (23), and the effect of immune checkpoint blockade is currently being evaluated in ongoing clinical trials. Future directions steer the path to validating prognostic and predictive biomarkers and analyzing treatment resistance mechanisms. 

## 7. Conclusions

The incidence of lung NENs is rising, possibly because of advanced radiological techniques. However, the incidence of SCLC has been steadily decreasing, likely due to declining smoking habits and other environmental factors. On the other hand, the survival rate of SCLCs and TCs has increased in recent years, likely due to a combination of earlier diagnosis and novel therapeutic strategies. The heterogeneity and rarity of the lung NENs make the diagnosis and management challenging, and owing to the rarity of the lung NENs other than SCLC, prospective trials are challenging to conduct. With the advent of newer treatments such as targeted therapy and immune checkpoint blockade, the treatment landscape of lung NENs is changing dramatically. Patients should be encouraged to participate in clinical trials whenever possible.

## Figures and Tables

**Table 1:** Demographic and clinical characteristics of patients with lung NENs.

	SCLC	LCNEC	TC	AC	Other	*p*-Value	
**Count, N (%)**	103,890 (83)	3303 (3)	8146 (6)	656 (1)	8974 (7)		
**Age, median (range in years)**	67 (15–99)	66 (18–94)	60 (10–96)	62 (12–90)	67 (12–99)	<0.01	
**Race**	*NHW (%)*	86,251 (83)	2595 (79)	6648 (82)	528 (80)	7009 (78)	<0.01
	*NHB (%)*	8804 (9)	397 (12)	560 (7)	46 (7)	912 (10)	
	*Hispanic (%)*	4503 (4)	168 (5)	688 (8)	57 (9)	606 (7)	
	*Other (%)*	4332 (4)	143 (4)	250 (3)	25 (4)	447 (5)	
**Sex**	*Male (%)*	53,661 (52)	1849 (56)	2690 (33)	236 (36)	4489 (50)	<0.01
	*Female (%)*	50,229 (48)	1454 (44)	5456 (67)	420 (64)	4485 (50)	
**Staging**	*Localized*	5810 (6)	659 (20)	6021 (74)	309 (47)	1420 (16)	<0.01
	*Regional*	23,875 (23)	869 (26)	1390 (17)	215 (33)	1680 (19)	
	*Distant*	74,205 (71)	1775 (54)	735 (9)	132 (20)	5874 (65)	
**Insurance**	*Not insured (%)*	1640 (2)	72 (2)	105 (1)	11 (2)	182 (2)	<0.01
	*Insured (%)*	40,742 (39)	2260 (68)	3879 (48)	491 (75)	4628 (52)	
	*Unknown (%)*	61,508 (59)	971 (30)	4162 (51)	154 (23)	4164 (46)	

NHW—non-Hispanic White; NHB—non-Hispanic Black.

**Table 2:** Multivariable Cox proportional hazards regression model highlighting OS and DSS in lung cancer patients.

Variable	OS Hazard Ratio	OS 95% Confidence Interval (CI)	DSS Hazard Ratio	DSS 95% Confidence Interval (CI)
**Stage (II) vs. Stage (I)**	1.25	1.23–1.27	1.28	1.26–1.31
**Stage (III/IV) vs. Stage (I)**	1.46	1.44–1.49	1.54	1.51–1.57
**Regional vs. Localized**	1.56	1.54–1.57	1.89	1.86–1.91
**Distant metastasis vs. Localized**	3.24	3.21–3.28	4.12	4.07–4.17
**Female vs. Male**	0.81	0.80–0.81	0.83	0.82–0.83
**TC vs. Adenocarcinoma**	0.30	0.28–0.31	0.16	0.15–0.17
**AC vs. Adenocarcinoma**	0.55	0.48–0.62	0.52	0.45–0.60
**SCLC vs. Adenocarcinoma**	1.11	1.10–1.12	1.11	1.10–1.12
**LCNEC vs. Adenocarcinoma**	1.15	1.11–1.19	1.15	1.11–1.20
**Race** **Hispanic vs. NHW** **NHB vs. NHW** **Other vs. NHW**	0.97	0.96–0.98	0.96	0.95–0.97
	1.01	1.00–1.02	1.00*	0.99–1.01 *
	0.80	0.79–0.81	0.79	0.78–0.80
**Single vs. Married**	1.17	1.17–1.18	1.15	1.15–1.16
**Uninsured vs. Insured**	1.22	1.19–1.25	1.19	1.16–1.23
**Rural vs. Urban**	1.08	1.07–1.09	1.08	1.07–1.09

*p*-value < 0.01; * except for DSS in NHB vs. NHW *p* = 0.961; AC—atypical carcinoid; TC—typical carcinoid; SCLC—small-cell lung cancer; LCNEC—large cell neuroendocrine carcinoma; NHW—non-Hispanic White; NHB—non-Hispanic Black.

## Data Availability

The data presented in this study are openly available on: https://seer.cancer.gov/data/, (accessed on 7 April 2021).

## Supplementary Materials

The following are available online at https://www.mdpi.com/article/10.3390/cancers13081753/s1 Table S1: (A) Multivariable Cox proportional hazards regression model highlighting OS change over time based on 5 years comparison among patients with SCLC. (B) Multivariable Cox proportional hazards regression model highlighting OS change over time based on 5 years comparison among patients with LCNEC. (X) Multivariable Cox proportional hazards regression model highlighting OS change over time based on 5 years comparison among patients with TC. (D) Multivariable Cox proportional hazards regression model highlighting OS change over time based on 5 years comparison among patients with AC.
