# Supplementary material for: Incidence and Survival Outcomes in Patients with Lung Neuroendocrine Neoplasms in the United States
Source: Cancers (Basel). 2021 Apr 7;13(8):1753. doi: 10.3390/cancers13081753 (PMC8067543; doi:10.3390/cancers13081753)
Supplement: Supplementary file 1 [file cancers-13-01753-s001.pdf]

(A) Supplementary Table 1SCLC

Variable	OS Hazard ratio	OS 95% Confidence Interval (CI)	p-Value
**1988–1992 vs. 2013–2015**	1.25	1.19–1.30	< 0.01
**1993–1997 vs. 2013–2015**	1.19	1.14–1.24	< 0.01
**1998–2002 vs. 2013–2015**	1.07	1.03–1.11	< 0.01
**2003–2007 vs. 2013–2015**	1.02	0.98–1.05	0.365
**2008–2012 vs. 2013–2015**	1.01	0.99–1.03	0.297

(B) LCNEC

Variable	OS Hazard ratio	OS 95% Confidence Interval (CI)	p-Value
**1998–2002 vs. 2013–2015**	1.09	0.87–1.37	0.471
**2003–2007 vs. 2013–2015**	0.96	0.81–1.14	0.635
**2008–2012 vs. 2013–2015**	1.01	0.92–1.12	0.831

(C) TC

Variable	OS Hazard ratio	OS 95% Confidence Interval (CI)	p-Value
**1988–1992 vs. 2013–2015**	2.28	1.53–3.38	< 0.01
**1993–1997 vs. 2013–2015**	2.35	1.62–3.40	< 0.01
**1998–2002 vs. 2013–2015**	1.54	1.09–2.18	0.015
**2003–2007 vs. 2013–2015**	1.30	0.97–1.74	0.082
**2008–2012 vs. 2013–2015**	1.03	0.83–1.27	0.810

(D) AC

Variable	OS Hazard ratio	OS 95% Confidence Interval (CI)	p-Value
**1998–2002 vs. 2013–2015**	1.39	0.58–3.36	0.461
**2003–2007 vs. 2013–2015**	0.85	0.43–1.71	0.652
**2008–2012 vs. 2013–2015**	1.24	0.84–1.81	0.280

Table S1. (A) Multivariable Cox proportional hazards regression model highlighting OS change over time based on 5 years comparison among patients with SCLC. (B) Multivariable Cox proportional hazards regression model highlighting OS change over time based on 5 years comparison among patients with LCNEC. (X) Multivariable Cox proportional hazards regression model highlighting OS change over time based on 5 years comparison among patients with TC. (D) Multivariable Cox

proportional hazards regression model highlighting OS change over time based on 5 years comparison among patients with AC.
